# Depression in Children and Adolescents with Autism Spectrum Disorder

**DOI:** 10.3390/children5090112

**Published:** 2018-08-21

**Authors:** Melissa DeFilippis

**Affiliations:** Department of Psychiatry, University of Texas Medical Branch, Galveston, TX 77555, USA; msdefili@utmb.edu

**Keywords:** autism spectrum disorder, depression, adolescents, prevalence, treatment

## Abstract

Autism spectrum disorder (ASD) has a high rate of psychiatric comorbidity. The prevalence of comorbid depression seems to correlate with higher functioning forms of ASD and increasing age. Adolescence is a time when youth struggle with identity and interpersonal relationships, and a diagnosis of ASD further complicates this process. Adolescents with ASD may be more aware of the social communication deficits that come with the diagnosis than children with ASD, and it is theorized that higher functioning adolescents may experience this more acutely. While this may be true, the lack of reliable rating and diagnostic scales for depression in individuals with ASD makes it difficult to accurately measure rates of depression among individuals with more severe verbal deficits. While some research has focused on the prevalence of comorbid depression in children and adolescents with ASD and on the associated risk factors, there is very little evidence guiding treatment, including no empirical studies on psychopharmacology for depression in this population. Available evidence exists only in psychosocial approaches to treatment at this time and is mostly limited to adult studies. Current evidence will be presented in this review, including prevalence rates of depression in youth with ASD, various risk and protective factors, the use of diagnostic rating scales, and treatment studies. The lack of evidence supporting various treatment approaches will be highlighted, including challenges specific to the treatment of depression in ASD, which are not addressed in the current treatment studies in typically developing youth with depression.

## 1. Introduction

Autism is a diagnosis that includes social communication deficits and restricted and repetitive behavior, interests, or activities, which are severe enough to significantly impair functioning [1]. The prevalence of autism spectrum disorder (ASD) has increased significantly over the past few decades, now affecting 1 in 59 children in the United States, based on recent Centers for Disease Control data [2]. ASD also frequently includes associated behavioral symptoms and comorbid psychiatric diagnoses, which can be as impairing as the core symptoms in some cases. It can be difficult to distinguish associated symptoms of ASD from comorbid psychiatric diagnoses (repetitive behaviors in ASD may be very similar to compulsions seen with obsessive-compulsive disorder, for example), making it hard to measure true comorbidity in youth with ASD. There is a relative lack in validated instruments for measuring comorbid psychiatric illnesses in the ASD population; therefore, it is no surprise that prevalence estimates of depression vary widely, from 2% to 30% [3]. Correctly identifying comorbid diagnoses in children and adolescents with ASD is important in guiding clinicians toward more appropriate and targeted treatment for these symptoms.

Depression is a fairly common diagnosis in youth, affecting about 12% of adolescents in the general population [4]. Most youth with one episode of depression will experience a recurrence of their symptoms at some point in adolescence or young adulthood [5]. Depression can include isolation and interpersonal struggles, which may also be present in ASD, and there is always a concern about suicidality in youth with depression. Individuals with ASD and depression may experience an increase in obsessions and rituals or, by contrast, a complete loss of interest in former preoccupations. Agitation, stereotypical behaviors, and self-injury may increase with depression. In patients with significant deficits in verbal skills, clinicians may need to rely more on vegetative signs, including decreased adaptive functioning skills and significant appetite and sleep disturbances compared to baseline [6]. Reviewing research on the prevalence rates and risk factors of depression in youth with ASD is important to help clinicians more accurately diagnose depression in this population and provide appropriate interventions. More research is needed examining efficacy and safety of treatment of depression in youth with ASD as current evidence is lacking in this area, and most treatment decisions are extrapolated from research in depressed youth with typical development [7].

## 2. Prevalence

Several studies examining prevalence of comorbid psychiatric symptoms and diagnoses in ASD have shown increased rates of both when compared to the general population, though the rates vary between studies. Accurately measuring the presence of depressive disorders in individuals with ASD is further complicated by the lack of appropriate rating and diagnostic scales for this population. In higher functioning youth with ASD, rating scales used in youth with typical development may be considered, but questions focused on more subjective symptoms, such as guilt and worthlessness, may be difficult for individuals with ASD and their parents to answer correctly [8]. In lower functioning individuals with ASD, scales developed for use in individuals with intellectual disability may be considered, such as the Reiss Scale [9] or the Disability Assessment Schedule [10]. The Autism Comorbidity Interview—Present and Lifetime version (ACI-PL) may be a more reliable measure in youth with ASD [11]. This scale includes questions about increased agitation, self-injury, and temper outbursts as well as asking about baseline functioning and measuring change from baseline. It also seeks to distinguish impairment from comorbid psychiatric diagnoses from impairment from the core ASD symptoms.

A population-based study examined the prevalence of psychiatric disorders in 112 children and adolescents (aged 10 to 14) with ASD [12]. About 70% of the youth had at least one comorbid disorder and 41% had two or more comorbid disorders based on parent interview using the Child and Adolescent Psychiatric Assessment (CAPA) [13]. The three-month point prevalence for depressive symptoms in this sample was 1.4%. A study of comorbid psychiatric diagnoses in ASD used the Autism Interactive Network database and included 4343 children and adolescents with ASD, 11% of which had a parent-reported history of comorbid depression [14].

A study examined the prevalence of comorbid psychiatric disorders in 177 youth with ASD, aged 3 to 18 years [15]. Using the Child Behavior Checklist, parents reported a 26% comorbidity rate of significant affective symptoms in their children [16,17]. Teacher ratings were lower, with only 6.2% rated at clinically significant ranges for affective symptoms, suggesting environmental effects on symptom expression. Other studies have shown different trends in multi-informant reports. One study examining comorbid psychiatric symptoms in 260 adolescents aged 11 to 17 years (including 43 with a diagnosis of Asperger syndrome or autism) showed higher rates of depressive and anxiety symptoms in the youth with ASD (26% in ASD youth compared to 12% in control group) [18]. Unlike the Kanne study, this one showed higher teacher-reported ratings of symptoms when compared to parental reports.

A validity/reliability study of the Autism Comorbidity Interview—Present and Lifetime version (a modification of the Kiddie Schedule for Affective Disorders and Schizophrenia—K-SADS) showed high rates of comorbid psychiatric disorders in a sample of 109 youth with ASD (aged 5 to 17 years) [12,19,20]. In this sample, 10% met diagnostic criteria for major depressive disorder, and 24% had subsyndromal depressive symptoms. A small study of 33 adolescents with Asperger syndrome (aged 12 to 17 years) showed 9% scoring in the significant range for depression on the Children’s Depression Inventory (CDI); however, 70% of the study participants were already being treated with antidepressants [21,22]. Prevalence studies of depression in youth with ASD are listed in Table 1.

A number of studies have compared prevalence rates of psychiatric disorders between youth with ASD and either the general population or typically developing youth. A large population-based longitudinal study included 6091 adolescents with and without ASD and examined the association with depression over eight years [23]. Using the Short Mood and Feelings Questionnaire (SMFQ) as the measure for depressive symptoms, adolescents with ASD had higher average scores when compared to the general population at age 10 years, and scores remained elevated in an upward trajectory until age 18 [24]. Depression at age 18 was associated with social communication impairments and bullying. Youth with ASD showed higher rates of depressive and anxiety symptoms than youth in the general population as measured by the Revised Ontario Child Health Study (OCHS-R), a revision of the CBCL [25,26]. This study included 59 children and adolescents with ASD and 1751 children from the community (general population control), aged 9 to 14 years. In the ASD group, about 17% scored in the significant rage on depression items compared to 3% in the general population.

A study of 70 adolescents, with mean age of 14, compared rates of depression in youth with ASD (*n* = 35) and youth without ASD (*n* = 35) [27]. Depressive symptoms were measured using the Centre for Epidemiological Studies Depression Scale—Children’s Version (CES-DC), and the results showed significantly higher rates of depressive symptoms in the ASD group versus the comparison group (66% and 40%, respectively) [28]. One study compared rates of depressive symptoms between 30 youth with ASD, 30 typically developing (TD) youth with major depressive disorder (MDD), and 35 typically developing youth without MDD, aged 7 to 17 years [29]. Depressive symptoms were assessed using the CDI and the Children’s Depression Rating Scale-Revised (CDRS-R) [30]. The ASD group showed significantly higher rates of depressive symptoms when compared to the TD group and similar rates of depressive symptoms as the MDD group. Depressive symptoms were associated with poorer global functioning in the ASD group.

Comparisons between ASD and other disorders have also shown differences in rates of comorbid psychiatric diagnoses and symptoms. A comparison study examined the difference in psychopathology between children and adolescents with autism and intellectual disability (ID) [31]. The ASD group had participants both with and without ID, but the ID group did not include participants with ASD. This study included 962 youth, aged 4 to 18 years, and used parent-reported symptoms on the Developmental Behavior Checklist (DBC-P) [32,33]. There were significantly higher levels of psychopathology, including depressive symptoms, in the ASD group when compared to the ID group. Higher scores on the depression scales were associated with increased age and higher intelligence quotient (IQ). Comorbid depression is commonly seen with other disorders, including disruptive behavior disorders. Youth with ASD were shown to have similar rates of depressive symptoms as youth with conduct disorder (CD) in a small study of 40 adolescents, aged 11 to 19 years [34].

## 3. Risk and Protective Factors

Youth with ASD and comorbid depression have a higher incidence of family history of depression when compared to youth with ASD without comorbid depression, similar to research in depressed youth without ASD [35]. Additionally, rates of depression are higher in first degree relatives of individuals with ASD when compared to first degree relatives of individuals with Down syndrome, when controlling for stress associated with raising a child with ASD [36]. Environmental stressors also play a role in the risk of depression in individuals with ASD, just as they do in typically developing youth. Youth with ASD have reported increased negative life events 12 months prior to the onset of depression when compared to controls [37]

Evidence shows risk of depression increases with increasing IQ and higher levels of functioning as well as with increasing age in youth with ASD. A large study examined prevalence and risk factors of anxiety and depression in 627 youth with autism, ages 1 to 17 years [38]; IQ ranged from 16 to 146. The parent-rated Pediatric Behavior Scale (PBS) was used to measure rates of anxiety and depressive symptoms in the study participants [39]. Prevalence of depressive symptoms ranged from 22% to 72% across the age groups, with the highest rates seen in the older groups. Along with age, the biggest predictors for depression in this study included increased verbal IQ and increased maternal-rated ASD symptom severity, with the latter being the single best predictor of both anxiety and depression.

A study of 1390 children and adolescents, including 350 with ASD, examined prevalence rates of several symptoms in different groups of children and adolescents, including controls in the general population and clinically referred youth with various psychiatric diagnoses, aged 6 to 16 years [40]. With regards to ASD and depression, the study showed higher rates of depressive symptoms (54%) among youth with ASD and IQ ≥80 when compared to youth with ASD and IQ <80 as measured by the PBS [39]. The study also found that youth with ASD and depression had lower rates of suicidal ideation and suicide attempts when compared to typically developing youth with depression. Though depression risk increases with age, evidence shows younger children with ASD are still at risk of developing symptoms. A study of 101 children with ASD, aged 4 to 9 years, showed a prevalence of depression that ranged from 6% (IQ < 70) to 19% (IQ ≥ 70) [41]. Though higher IQ was associated with a higher prevalence of depressive symptoms, this finding did not reach statistical significance in this study. Anxiety symptoms, however, were significantly associated with a higher IQ.

A study examined the effect of different coping strategies and level of social functioning on depression in 120 male subjects (mean age 11), including 63 youth with ASD (IQ ≥ 80) and 57 youth with typical development [42]. When compared to the youth with typical development, those in the ASD group who reported using avoidant strategies to manage stressful situations reported fewer symptoms of depression on the CDI, suggesting a possible adaptive coping strategy in this group. Poor social functioning was associated with increased symptoms of depression [22].

The trajectory of depressive symptoms in youth with ASD has been studied with some mixed results; however, all evidence supports higher rates of depression than in the general population that remains into adulthood. One study showed improvements of depressive symptoms in youth with ASD as they aged [43]. Other clinical trials, however, have shown symptoms of depression either remaining elevated at similar rates or at increasing rates in older adolescents and young adults with ASD [23,44]. Social communication deficits have been shown to be associated with an increased risk of self-harm and suicidal ideation in youth [45]. This risk appears to be independent of an ASD diagnosis, suggesting typically developing youth with social communication deficits are also at an increased risk of suicidal thoughts and behaviors. Depression in early adolescence appears to contribute somewhat to this risk, which highlights the importance of early interventions for depression in children and adolescents. More research is needed to better understand risk factors associated with suicidal thoughts and behaviors in youth with ASD.

## 4. Treatment

Treatment for depression in youth with ASD tends to be guided by evidence from studies of typically developing children and adolescents with depression. There is not much evidence to help clinicians make treatment decisions, and there are no randomized, placebo-controlled trials examining efficacy of antidepressants for the treatment of depression in youth with ASD [46]. Antidepressant treatment studies in youth with ASD have focused almost exclusively on the core symptoms of autism, specifically repetitive behaviors. Randomized controlled trials have failed to show efficacy of selective serotonin reuptake inhibitors (SSRIs) for repetitive behaviors in youth with autism, and side effects have been a concern in these studies (specifically, serotonergic activation effects) [47,48]. A small, 10-week, open-label study of escitalopram in 28 children and adolescents with ASD (aged 6 to 17 years) showed significant improvement in symptoms of irritability but did not measure depressive symptoms specifically [49]. The youth in the study experienced dose-related adverse effects of irritability and hyperactivity, and a quarter could not tolerate doses above 10 mg. Responders tended to do so at lower doses of escitalopram.

The lack of evidence supporting the use of SSRIs in children and adolescents with ASD is concerning as these are the most commonly prescribed psychotropic medications in individuals with ASD [50,51]. A study examining medication use in 286 adolescents and adults with ASD (mean age 21) showed 33% of the study participants were prescribed antidepressants, rising to 43% by the end of the four-year study [52]. This was the most common psychotropic prescribed to the individuals in the study. A Cochrane Review concluded that antidepressants should only be used on a case-by-case basis for depression or obsessive-compulsive disorder in children with autism, but the evidence does not really support its use, and there is significant evidence showing potential harm in this patient population [53]. Clinicians should use their clinical judgment when considering antidepressants for children and adolescents with ASD. If the decision to treat with antidepressants is made, then clinicians should choose low doses to start, titrate slowly, and carefully monitor for response and adverse effects.

Much of the evidence pertaining to psychosocial interventions, such as cognitive behavior therapy, is focused on treating either core symptoms of ASD, associated behavioral symptoms, or anxiety [54]. A few studies have examined the efficacy of psychosocial interventions for depression in individuals with ASD; however, most of these have been adult studies. One study examined the efficacy of group cognitive behavioral therapy on symptoms of anxiety, stress, and depression in 32 adolescents and young adults with ASD (aged 15 to 25 years) [55]. When compared to the wait list control, individuals in the CBT group with symptoms of depression pretreatment showed significant improvement in their depressive symptoms, as measured by the Depression Anxiety Stress Scales (DASS) [56]. Studies in adults with ASD have shown mindfulness-based therapy and social and vocational skills training to be effective for symptoms of depression, but neither intervention has been studied as a treatment for depressive symptoms in children or adolescents with ASD [57,58]. Evidence is still lacking in this area but psychosocial interventions, such as cognitive behavior therapy, should be considered as treatment options for youth with ASD and comorbid depression.

## 5. Conclusions

Depression is a fairly common comorbid diagnosis in children and adolescents with autism. It may be harder to recognize in this patient population due to communication deficits, and symptoms of depression may compound the interpersonal difficulties that youth with ASD already experience. Accurate diagnosis may be difficult, especially in children and adolescents with limited verbal skills. Reliance on parental-report and change from baseline behaviors is very important in these cases. Current diagnostic scales may not be ideal for diagnosing depression in youth with ASD, and scales developed specifically for youth with ASD, such as the ACI-PL, may be more reliable in this patient population. Research examining the efficacy of various treatments for depression in individuals with ASD is very limited, especially in children and adolescents, and there are currently no randomized controlled trials examining the safety and efficacy of treatments for depression in this population. Considering the rates of depression seen in youth with ASD are higher than those observed in typically developing youth, the lack of guidance for clinicians treating these patients is concerning. Additionally, evidence suggests youth with ASD are at a higher risk of developing adverse effects with antidepressant medications, and traditional psychosocial interventions need more study to determine whether they are as effective in this population as in typically developing youth with depression. Antidepressants continue to be the most commonly prescribed psychotropic medications for patients with ASD despite concerns about side effects and lack of evidence supporting their use in this patient population. Future research should focus on symptom expression in depressed youth with ASD and how it may differ from typically developing youth. It should also focus on establishing appropriate assessments for these symptoms and safe and effective treatments for depression in this patient population.

## Figures and Tables

**Table 1:** Prevalence Studies of Depression in Youth with autism spectrum disorder (ASD).

Study/Year	Number of Participants	Prevalence of Depression	Scale Used	Comments
Simonoff et al., 2008	112	3 month point prevalence = 1.4%	CAPA	
Rosenberg et al., 2011	4343	11%	Parent-reported community diagnoses of depression	Autism Interactive Network Database
Kanne et al., 2009	177	26%—parent-rated 6%—teacher-rated	CBCL	
Hurtig et al., 2009	260	26% (anxiety or depressive symptoms)	CBCL	
Leyfer et al., 2006	109	10%—MDD 24%—subsyndromal depressive symptoms	ACI-PL	Scale designed specifically for measuring psychiatric comorbidity in ASD
Barnhill et al., 2001	33	9%	CDI	70% of participants already on antidepressant medication
